# Recent Advancements in Drug Delivery of Sinomenine, A Disease-Modifying Anti-Rheumatic Drug

**DOI:** 10.3390/pharmaceutics14122820

**Published:** 2022-12-16

**Authors:** Xin Chen, Chengcheng Lu, Yanwen Duan, Yong Huang

**Affiliations:** 1 Xiangya International Academy of Translational Medicine, Central South University, Changsha 410013, China; 2 Hunan Engineering Research Center of Combinatorial Biosynthesis and Natural Product Drug Discovery, Changsha 410013, China; 3 National Engineering Research Center of Combinatorial Biosynthesis for Drug Discovery, Changsha 410011, China

**Keywords:** rheumatoid arthritis, sinomenine, half-life, side effect, drug delivery, drug release behaviors

## Abstract

Sinomenine (SIN) is a benzyltetrahydroisoquinoline-type alkaloid isolated from the dried plant root and stem of *Sinomenium acutum* (Thumb.) Rehd.et Wils, which shows potent anti-inflammatory and analgesic effects. As a transforming disease-modifying anti-rheumatic drug, SIN has been used to treat rheumatoid arthritis over twenty-five years in China. In recent years, SIN is also in development for use against other disorders, including colitis, pain, traumatic brain injury, and uveitis. However, its commercial hydrochloride (SIN-HCl) shows low oral bioavailability and certain allergic reactions in patients, due to the release of histamine. Therefore, a large number of pharmaceutical strategies have been explored to address these liabilities, such as prolonging release behaviors, enhancing skin permeation and adsorption for transdermal delivery, targeted SIN delivery using new material or conjugates, and co-amorphous technology. This review discusses these different delivery strategies and approaches employed to overcome the limitations of SIN for its efficient delivery, in order to achieve improved bioavailability and reduced side effects. The potential advantages and limitations of SIN delivery strategies are elaborated along with discussions of potential future SIN drug development strategies.

## 1. Introduction

As a chronic autoimmune joint disorder, rheumatoid arthritis (RA) affects about 1% of the world’s population [1]. Like many other autoimmune diseases, there is a significantly higher risk of RA in women than men and the risk increases with age. These patients typically suffer joint tenderness and swelling, leading to destruction of synovial joints, which eventually results in impaired movement and disability [2]. Non-steroidal anti-inflammatory drugs (NSAIDs) are commonly prescribed; however, their long-term use may cause heart attacks and strokes. As effective immunomodulatory and immunosuppressive drugs, diseases-modifying antirheumatic drugs (DMARDs), including sulfasalazine, leflunomide, hydroxychloroquine, methotrexate, and biologics, such as adalimumab, abatacept, certolizumab pegol, etanercept, golimumab, infliximab, rituximab, and tocilizumab, are used to treat RA with distinct mechanisms of action. The caveat is that the wide-spread use of biologics is associated with much higher price tags, which may prevent their wide use in developing countries [2,3,4].

**Figure 1:**
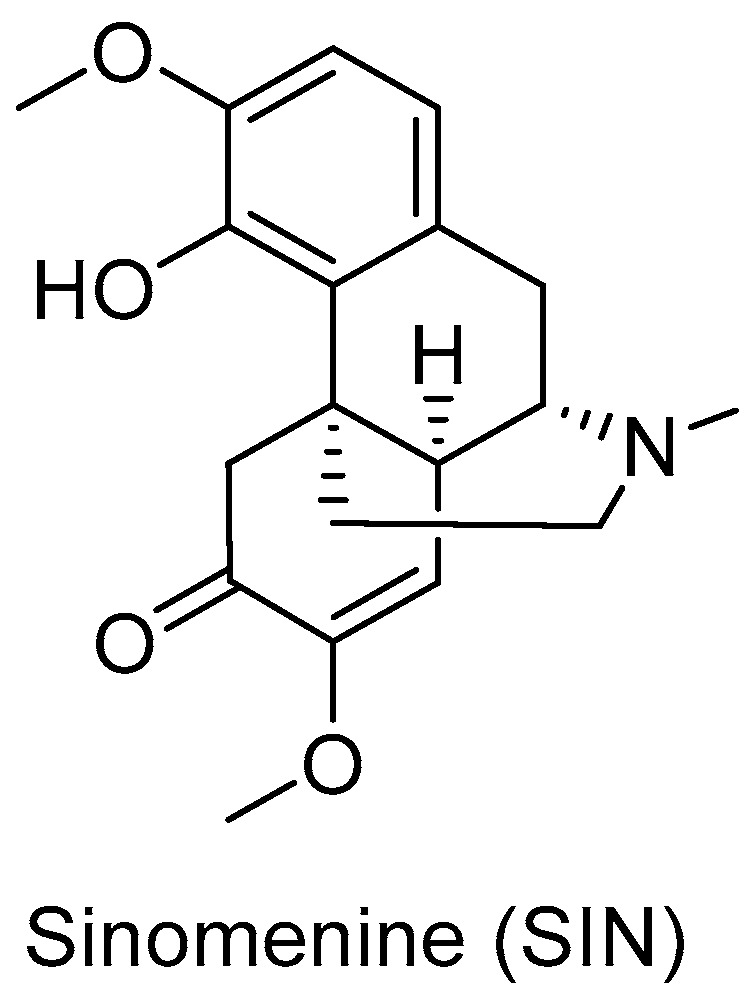
The structure of SIN.

Sinomenine (SIN) is a benzyltetrahydroisoquinoline-type alkaloid isolated from the traditional Chinese medicine *Sinomenium acutum* (Thunberg) Rehder & E.H. Wilson (family Menispermaceae Juss), whose dried root and stem have been traditionally used to treat RA in China [5]. SIN was listed into the Chinese Pharmacopeia (ChP) 2005 and its hydrochloride (SIN-HCl) in the forms of tablets or injection has been used for RA treatment for years (Figure 1). In a meta-analysis of eligible clinical studies involving 1,500 RA patients, SIN showed superior clinical efficacy and fewer adverse events compared to methotrexate [6]. In particular, SIN could be used in a combination therapy with methotrexate in a randomized controlled clinical trial, showing comparable ACR50 response with methotrexate and leflunomide at week 24, while the former combination showed significant reductions of liver toxicity and gastrointestinal adverse reactions in patients [7].

Although the underlying mechanism of SIN in RA therapy remains to be established, many reports suggested that SIN effectively regulates the immune responses of T cells and Th cells, as well as the production and secretion of inflammatory cytokines in many cell types and animal models, as well as RA patients [8,9,10,11,12]. For example, SIN regulates the secretion of inflammatory cytokines and monocyte/macrophage subsets [11] and the expression of bone marrow differentiation primary response protein 88 [13]. The inhibition of activated TLR4/NF-κB signaling pathway [14] is also reported. In addition, SIN may up-regulate tissue inhibitors of metalloproteinases by inhibiting α7 nicotinic acetylcholine receptor [15,16,17], microsomal prostaglandin E synthase 1 [12], metalloproteinases, and proinflammatory cytokines [18]. SIN can also activate the p62^Thr269/Ser272^-Keap1-Nrf2 feedback loop [19]. Due to its significant immunomodulating activity for RA, SIN was thus suggested to be a promising and affordable DMARD [11]. Furthermore, SIN also possess a variety of other promising activities, including analgesic [20], anti-inflammatory [21], antitumor [22], cardiovascular [23], pulmonary protective [24], neuroprotective [25], and other activities [5,25,26], suggesting its great potential to be repurposed against multiple diseases.

However, SIN-HCl exhibits a rather short half-life and rapid metabolism [27,28]. The low oral bioavailability promotes its frequent drug administration to achieve desirable therapeutic effects. SIN-HCl also promotes the release of histamine, which leads to various side effects, e.g., allergic and gastrointestinal reactions [29]. In addition, as a water-soluble drug with a short half-life, its rapid release and clearance *in vivo* will lead to a huge fluctuation of drug–plasma concentrations, thereby affecting its efficacy and increasing the likelihood of side effects.

Therefore, there is great interest to develop novel drug delivery routes or solid formulations of SIN with sustained-release behaviors. A large number of pharmaceutical strategies have been explored to address the above liabilities, including the development of tablets or co-amorphous solid formulations with sustained release behaviors, enhanced skin permeation and adsorption for transdermal delivery, targeted SIN delivery using new material or conjugates, and other delivery systems. This review discussed these different delivery strategies and approaches employed to overcome the limitations of SIN for its efficient delivery, in order to achieve improved bioavailability and reduced side effects. The potential advantages and limitations of SIN delivery strategies are discussed.

## 2. Oral Delivery Systems

**Figure 2:**
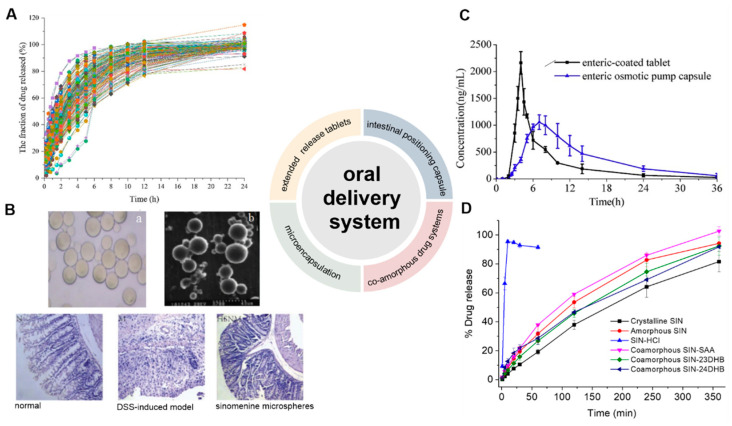
Development of oral administration systems of SIN. (**A**) Dissolution profiles of 36 batches of SIN-HCl sustained-release tablets showing complete drug release with a zero-order kinetics (*n* = 5) [30]. The tablets consisted of with SIN-HCl, osmotic agents, and polymers for promoting permeation. (**B**) Light (**a**) and electron microscope (**b**) images of SIN microspheres, which exhibited improved treatment for DSS-induced mouse models by Hematoxylin–Eosin (H&E) staining of colon sections in different treatment groups [10]. (**C**) Plasma concentration–time curves of SIN from both enteric osmotic pump capsules or enteric-coated tablets in beagle dogs after oral administration (*n* = 6) [28]. (**D**) Release profiles of SIN from crystalline SIN, amorphous SIN, SIN-HCl, as well as co-amorphous SIN and three different phenolic acids [37]. (Reprinted with permission form Refs. [10,28,30,37]).

Oral drug formulations are the most widely used drug forms because of their ease of manufacture, transportation, storage, and use. There are currently thirteen SIN-HCl solid formulations, including ordinary tablets, sustained release tablets, enteric-coated tablets, and capsules. As the marketed sustained release formulation is 1–2 tablet (s) (60 mg each) twice daily, longer sustained release strategies for SIN are needed (Figure 2).

### 2.1. Extended-Release Tablets

The development of extended-release drug tablets is a common technique to lengthen the short half-life of drugs and mitigate the risk of toxic and side effects caused by drastic fluctuations in blood drug concentration. The preparation of extended-release tablets of SIN-HCl could be optimized by varying the amount of SIN-HCl and drug excipient hydroxy propyl methyl cellulose, as well as manufacturing parameters, such as mixing time, pressure, and tablet yield [30]. The resulting SIN-HCl tablets can be completely released for 24 h (Figure 2A). A protection–graft–deprotection reaction was also used to prepare two degradable copolymers of poly (p-dioxanone) with different graft chain lengths and chitosan for the sustained release of SIN [31]. In both phosphate-buffered saline (PBS) and artificial gastric juice, SIN co-pressed with pure chitosan totally released within 4 and 5 h, while only 25.2% and 36.2% of SIN released when co-pressed with the copolymer with longer graft chain.

### 2.2. Microencapsulation

Microencapsulation refers to reservoir-type spherical particles formed by wrapping the drug as a capsule core, while microsphere is a skeletal particle formed by dissolving, dispersing, or adsorbing the drug into a carrier, such as polymers. Microencapsulation is an important technology in modern drug formulations for targeted drug delivery and controlled release, resulting in the reduction of drug irritation and improved drug stability. SIN-loaded microcapsules with biodegradable polylactic acid were prepared by a reverse-phase emulsification-in-liquid drying method with a drug encapsulation ratio and loading capability of 89.2% and 8.9%, respectively [32]. In simulated intestinal fluids, SIN showed a continuous and slow releasing behavior with 74% to 87% release in the presence of different amount of emulsifiers after 96 h. SIN-loaded polyelectrolyte multilayer microcapsules were prepared with a layer-by-layer encapsulation method, including polydimethyldiallyl ammonium chloride/alginate, gelatin/alginate, and chitosan/alginate microcapsules, which exhibited varying sustained release behaviors [33].

A surface molecular imprinting strategy was used to prepare SIN microspheres using β-cyclodextrin-grafted chitosan as the matrix and SIN as the template [34]. The resulting microspheres adsorb 55.9 mg/g of SIN, while 78% of SIN could be released in pH 7.4 after 24 h. A molecule-imprinted polymer using pyroxene as the matrix and SIN as the template showed a similar SIN adsorption rate of 57.4 mg/g [35]. The acrylate-coated SIN chitosan enteric-soluble microspheres showed its cumulative release of 8.91% and 59.52% in the simulated intestinal fluid at pH 6.8 and 7.4, respectively [10]. With the addition of mice colon contents in the above medium, the release rate of SIN further increased to 72.54%, suggesting their good colon-targeting properties. In the animal model of dextran sulfate sodium (DSS)-induced colitis, SIN enteric-coated microspheres-treated mice exhibited attenuated inflammatory factors in a dose-dependent manner (Figure 2B). This strategy also avoids the gastric irritation by reducing the frequency of SIN administration, which may eventually reduce blood-drug concentration fluctuation.

The sustained-release pellets of SIN-HCl, prepared by a novel whirlwind fluidized bed technology, were also recently reported [36]. The novel whirlwind fluidized bed exhibited a higher yield of pellets (>96%) and SIN-HCl encapsulation efficiency (>90%) compared to that of the conventional fluidized bed with the optimized operating parameters and formulation. The encapsulated SIN-HCl pellets exhibited sustained-release and stable dissolution behaviors among different batches over 12 h.

### 2.3. Intestinal Positioning Capsule

An enteric positioning osmotic pump capsule was developed [28]. The capsule shell was prepared by impregnation with cellulose acetate as the semi-permeable membrane, hydroxypropyl methacrylate phthalate HP50 for enteric localization, and polyethylene glycol 6000 as the toughening agent, while SIN-HCl, osmotic agent, and permeation promoting polymer were filled. This specifically designed capsule did not release SIN under acidic conditions, but completely released SIN within 14 h in an alkaline medium, indicating excellent intestinal targeting behavior. Further *in vivo* evaluation showed that the capsule had longer T_max_, extended average retention time, and more stable plasma concentration than tested commercial enteric-coated tablets (Figure 2C).

### 2.4. Co-Amorphous Drug Systems

Co-amorphous drug systems are a class of homogeneous amorphous pharmaceutical solid forms containing two or more low-molecular-weight compounds, many of which showed improved aqueous solubility, physical stability, and drug loading capacity. Several new SIN co-amorphous drug systems were recently designed and developed, in order to alleviate the possible side effects of SIN for RA treatment, using co-formers phenolic acids including salicylic acid, 2,3-dihydroxybenzoic acid and 2,4-dihydroxybenzoic acid, NSAIDs including indometacin, naproxen, and sulindac, antihistamine drug tranilast, antibiotics platensimycin, and sulfasalazine [37,38,39,40]. The formations of a homogenous co-amorphous systems were based on powder X-ray diffraction and temperature-modulated differential scanning calorimetry, while Fourier transform infrared spectroscopy, X-ray photoelectron spectroscopy, and NMR experiments revealed the formation of salts and strong intermolecular interactions between SIN and its co-formers in the respective drug systems. In dissolution experiments, different degrees of gelation behaviors among all the SIN co-amorphous systems were observed, thus leading to sustained release of SIN compared to the commercial SIN-HCl. SIN-HCl was totally released within 10 min in PBS, while SIN could not compliantly release from most of the co-amorphous systems after 6 h (Figure 2D).

## 3. Percutaneous Delivery Systems

**Figure 3:**
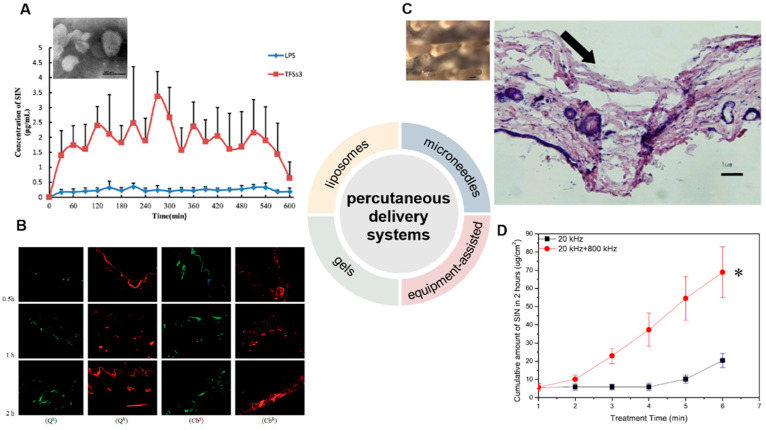
Development of percutaneous delivery systems of SIN. (**A**) The transmission electron microscope of monoterpene edge activated PEGylated transfersomes (×20,000) and the release of SIN after its application to the abdominal skin of rats (*n* = 5) [41]. (**B**) laser scanning confocal microscopy images of skin samples for sodium fluorescein-loaded cubic LC gel (Q^S^), rhodamine B-loaded cubic LC gel (Q^R^), sodium fluorescein-loaded carbomer gel (Cb^S^), and rhodamine B-loaded carbomer gel (Cb^R^) in Franz cells during different periods (×100) [44]. (**C**) The close-up views of microneedles and the methylene blue-stained frozen section of rat’s abdominal skins treated by microneedles [45]. The arrow means the pierced position. (**D**) Cumulative release of SIN under ultrasound with single- or dual-frequency treatment [48]. * *p* < 0.05. (Reprinted with permission from Refs. [41,44,45,48]).

The development of local percutaneous drug delivery systems can reduce the side effects of SIN and enhance patient compliance. Many percutaneous drug delivery systems have thus been developed, including liposomes [41,42], hydrogels [43,44], and microneedles [45]. In addition, some equipment-assisted percutaneous drug delivery methods were also used, such as electroporation and dual-frequency ultrasound [46,47,48] (Figure 3).

### 3.1. Liposomes

Liposomes have shown excellent biocompatibility and are often used for transdermal drug delivery. The local delivery of liposome-encapsulated SIN may enrich SIN in the target site and thus reduce the side effects caused by systemic absorption. Conventional liposomes prepared with soybean phospholipid, cholesterol, and vitamin E can be used to encapsulate SIN, while they still suffer limited permeability [49,50]. Ethosomes are a new type of liposomes containing a high concentration of alcohol inside [51], which would effectively deliver SIN through the stratum corneum to the deeper layers of skin, and even into the blood circulation [52]. The optimized SIN-loaded ethosomes (SE) with negative charges and diameter of 157.08 nm were prepared. The skin penetration and deposition of SIN in ethosomes were 663.8 and 18.5 μg/cm^2^ within 24 h, while those of SIN in ethanol-water solution were only 329.2 and 5.2 μg/cm^2^, respectively. These results indicated that SE could significantly improve the transdermal property of SIN. Skin irritation tests showed that ethosomes caused no skin rash and edema in rabbits within 72 h, suggesting excellent biocompatibility. In a xylene-induced mouse ear swelling model, SIN encapsulated ethosome had an inhibition rate of 30.01% in ear swelling, significantly higher than that treated by SIN-HCl ethanol-water solution (20.83%). Transfersomes (TFSs) are a class of elastic liposomes composed of phospholipids and edge activators; the addition of edge activators could interfere and deform the phospholipid bilayers of vesicles and lead to their deformability [53,54]. The resulting flexible membranes enable TFSs to be transported through the skin and bypass the cuticle barrier, thereby increasing drug deposition in the skin and prolonging the duration of effective drug concentration [55].

SIN-HCl TFSs were prepared using sodium deoxycholate as the edge activator, and SIN-HCl liposomes were used as a control [56]. The *in vitro* permeation experiment showed that the cumulative transdermal permeated amount of SIN-HCl from SIN-HCl TFSs was 1.7 times higher than that from SIN-HCl liposomes at 36 h. SIN-HCl TFSs showed about 8.8 and 8.0 times of *in vivo* steady-state blood concentration (C_ss_) and the area under the drug concentration–time curve from time zero to t (AUC_0–t_) compared to those of SIN-HCl liposomes in a skin pharmacokinetic investigation. In blood pharmacokinetic tests, SIN-HCl TFSs exhibited 3.7 and 2.9 times of C_ss_ and AUC_0–t_ compared to that of the control group. These data suggest that TFSs could effectively improve the transdermal absorption of SIN-HCl. A mixture of monoterpene edge-activated PEGylated transfersomes (MMPTs) was also used to improve the *in vivo* transdermal delivery efficiency of SIN [41] (Figure 3A). In the *in vitro* skin penetration test, the cumulative skin penetration of SIN in the optimized formulation TFSs3 was 1.5 and 3 times of those of sodium deoxycholate edge activated transfersomes (DTFS) and ordinary liposomes, respectively. Pharmacokinetic evaluation in rats revealed that the equilibrium concentration and area under the curve of SIN encapsulated in TFSs3 were 8.7 and 8.2 times of those in ordinary liposome, respectively, indicating more effective skin penetration of SIN. Confocal laser scanning microscopy and double-sited microdialysis coupled with LC-MS/MS were used to reveal the biodistribution of MMPTs in different cortex and the pharmacokinetic properties of SIN in blood and joint cavity, as well as intrinsic mechanisms of the local transdermal delivery [57]. The control liposomes only reached to the cuticle, while MMPTs reached to the deep cortex. The equilibrium concentration and area under the curve of SIN delivered by MMPTs in articular cavity were 2.1 and 2.5 times higher than that of control liposomes, respectively, while only about one third compared to that of control liposomes in blood. Taken together, MMPTs could efficiently deliver SIN into the deep cortex and subsequently be enriched in the joint cavity. Further delivery of SIN using the combination of the TFSs and ethanosomes resulted in a transsethosome (TE) for transdermal administration of SIN, featuring even more effectively delivery of SIN through the skin [42]. The surface of TE was also modified with the antioxidant ascorbic acid (AS-TE) to rebalance reactive oxygen species in the inflammatory microenvironment and achieve targeted drug delivery [58]. The TE and AS-TE had similar transdermal effects, which were about 7.6 times as large as that of SIN-HCl aqueous solution. Subsequent micro-dialysis on synovial fluid showed that the AS-TE group exhibited higher drug concentration in the synovial fluid of rabbits with RA than the TE group, indicating its excellent targeting ability to inflammatory microenvironment. In a rat RA model, AS-TE encapsulated SIN showed better therapeutic effect, which significantly reduced the symptoms of joint swelling after three weeks of treatment.

### 3.2. Gels

Gels belong to semi-solid materials with three-dimensional network structures and good biocompatibility, and are widely used in drugs, e.g., piroxicam gel, terbinafine hydrochloride gel, and ofloxacin gel, and various cosmetics [59]. An optimized pluronic lecithin organogel (PLO)-based SIN formulation was prepared, which showed a SIN infiltration rate of 146.55 ± 2.93 μg/cm^2^/h into the skin, higher than that of SIN-loaded carbomer gel (120.39 μg/cm^2^/h). In addition, more SIN was deposited into skin from PLO (10.08 ± 0.86 μg/cm^2^) than that from carbomer gel (6.01 ± 0.04 μg/cm^2^). Subsequent *in vivo* skin microdialysis studies revealed that PLO showed much higher SIN maximum concentration in permeation and drug-deposition studies (150.27 ± 20.85 and 67.95 ± 5.21 μg/mL) than that of carbomer gel (29.66 ± 1.50 and 6.73 ± 0.88 μg/mL). Cubic liquid crystal gels are useful for the controlled release of small molecules, proteins, peptides, and even nucleic acids, due to their stable thermodynamic properties and highly ordered internal structures [60,61]. These gels were used for the transdermal delivery of SIN, and showed increased cumulative release of SIN with the increase of SIN loading when evaluated in Franz diffusion cells using the rat ventral skin dermis oriented to the receiving chamber [62]. A double-loaded cubic liquid crystal gels containing cinnamaldehyde and SIN-HCl with pseudoplastic fluid behavior was further developed, in which cinnamaldehyde further enhances the transdermal delivery of SIN [44] (Figure 3B).

### 3.3. Microneedles

Microneedles combine the features of conventional injections and patches, and can be divided into solid, drug-coated, and drug-loaded dissolving microneedles for transdermal drug delivery [63,64,65]. The solid microneedle only punctures the skin to form a drug delivery channel, followed by the application of drugs for transdermal delivery. Drugs can be either coated on the surface of microneedles or embedded into soluble microneedles; the former are difficult in continuous drug administration, while the later ones and the embedded drugs are completely dissolved or degraded in the skin [66]. A SIN-loaded dissolving microneedles (SH-DM) composed of maltose and poly (lactic acid-glycolic acid) copolymer was prepared using casting method and shown good mechanical strength [67]. The SH-DM showed a higher cumulative permeability and faster penetration rate compared to the SIN gel (SH-G) group. Pharmacokinetic study revealed that SH-DM had a later peak time and larger maximum concentration (C_max_) of SIN than that of SH-G, which holds 1.99 times the area under the curve of SIN-HCl of that from SH-G. Similar SH-DM prepared with maltose and polyvinyl alcohol also exhibited ideal mechanical strength and better transdermal drug delivery behavior and bioavailability than the control hydrogel in both *in vitro* infiltration and pharmacokinetic experiments [45] (Figure 3C). New composite microneedles were casted with chondroitin sulfate and PVP, which integrated with phytriol/water system containing SIN-HCl [68]. The composite microneedles can achieve continuous SIN release in transdermal drug delivery, since it had longer peak time and a lower peak SIN concentration [69].

### 3.4. Physically Assisted Delivery Systems

Electroporation has been used for the transdermal delivery of small-molecule drugs by forming water channels in the stratum corneum through short high-voltage pulses and thus resulting in instantaneous penetration [70]. The electroporated transdermal delivery of SIN-HCl showed the highest C_SF_/C_plasma_ (SIN concentration in synovial fluid vs. that in plasma) among oral, intravenous, and electroporation transdermal delivery systems in a rabbit animal model [46]. In addition, the electroporation administration parameters, including frequency, waveform of exponential curve, and intensity of pulses could be optimized to improve delivery efficiency by 1.9- to 10.1-fold or 1.6- to 47.1-fold than that of the passive diffusion in mouse skin and miniature pig skin, respectively [47]. The concentration of SIN in synovial fluid reached to 20.84 ng/mL with the highest C_SF_/C_plasma_ after electroporation in observatory clinical trials, suggesting SIN-HCl could be effectively delivered to the site of lesions in patients with RA. Dual-frequency ultrasound (20 kHz + 800 kHz) was used for transdermal delivery of SIN-HCl, showing a significant higher cumulative penetration than that of each single frequency ultrasound (20 kHz or 800 kHz) and the sum of them [48] (Figure 3D).

## 4. Injection Delivery Systems

### 4.1. Intra-Articular Local Injections

**Figure 4:**
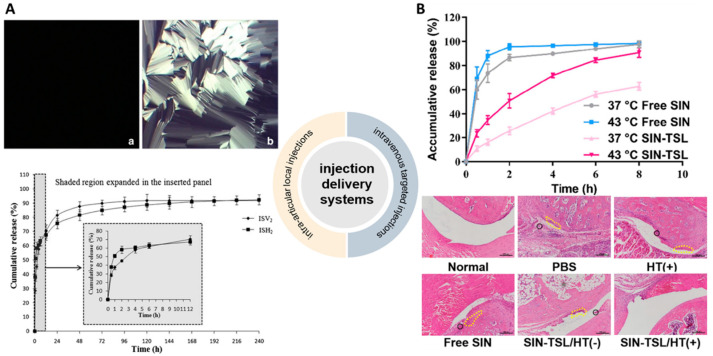
Development of injection delivery systems of SIN. (**A**) Images of cubic liquid crystalline gel (**a**) formed from ISV_2_ and hexagonal liquid crystalline gel (**b**) formed from ISH_2_ in excess water and the *in vitro* release profiles of 6 mg/g SIN-HCl from ISV_2_ and ISH_2_. [71]. (**B**) *In vitro* release of SIN and SIN-TSL in PBS (*n* = 3) and H&E staining of ankle joints among different treatment groups, where the inflammatory cell infiltration (black circle) and the bone erosion (dashed yellow) were marked [77]. (Reprinted with permission form Refs. [71,77]).

Since the marketed SIN-HCl injection exhibited strong histamine releasing effect, intra-articular local injection may enable SIN to reach the joint cavity to improve local drug concentration, which would reduce its dosage and systemic side effects [71,72,73,74]. Liquid crystal-based articular SIN-HCl delivery systems using low-viscosity precursors to form *in situ* cubic liquid crystal (ISV_2_) or *in situ* hexagonal liquid crystal (ISH_2_) achieved sustained release of SIN over 6 or 10 days, respectively [71] (Figure 4A). SIN-HCl-loaded ISH_2_ delivery systems were prepared using a liquid precursor mixture of phytantriol (PT), vitamin E acetate (VEA), ethanol (ET), and water. The optimized ISH_2_ formula with in a PT/VEA/ET/water mass ratio of 60.8:3.2:16.0:20.0 showed lower SIN C_max_ and AUC_0–∞_ in plasma compared to that of SIN-HCl normal saline solution in rat model, indicating its reduced leakage into the systemic circulation after intra-articular administration [75]. The treated rats with adjuvant induced arthritis had a residence of SIN for at least 168 h, which was significantly longer than the control group (48 h). In addition, the significant therapeutic effect of SIN-HCl-loaded ISH_2_ on synovial inflammation of the knee joints in rats was confirmed. Furthermore, the hexagonal liquid crystal encapsulated SIN could inhibit the inflammatory factor IL-Iβ at a low level within 12–72 h, while SIN solution only exerts its inhibitory effect within 0.25–2 h. An *in situ* cubic liquid crystal intra-articular injection system using glycerol monoleate as matrix also prolong the release of SIN up to 9 days in *in vitro* release experiments [72].

An injectable SIN-sodium hyaluronate sustained release system for intra-articular injection was developed to treat osteoarthritis in a rabbit model, resulting in a Mankin score of 0.73 ± 0.78, which was significantly lower than those from rats treated by saline, SIN-HCl solution, and sodium hyaluronate with a score of 4.93 ± 0.83, 2.57 ± 1.07, and 2.63 ± 0.96, respectively [76]. Pharmacokinetic study in joint cavity revealed that its AUC_(0–12h)_ and average retention time were 2.9- or 1.88-fold of that of SIN solution group, respectively, which eventually resulted in about two-fold improved therapeutic effects [73].

### 4.2. Intravenous Targeted Injections

In addition to intra-articular local injections, some intravenous targeted injections of SIN have also been reported. A thermosensitive liposome loaded with SIN exhibited cumulative release of less than 60% SIN at 37 °C after 8 h in PBS (pH 7.4), while the thermal oxidation of vinyl ether linkage at 43 °C led to more than 80% SIN release. This liposome could decrease the thickness of rat paws in a rat RA model, especially under microwave radiation, probably due to efficient delivery of liposomes in the lesion sites and the heat-responsive release of SIN from liposome nanoparticles [77] (Figure 4B).

Synovial-targeting linear and cyclic peptides were conjugated to SIN and the resulting conjugates containing the cyclic peptide were more stable than the one in mouse serum and inflammatory joint homogenates, which also showed better efficacy and tissue targeting behavior [78]. Prussian blue nanoparticles were employed to construct a biomimetic nanocomplex of SIN-HCl for overcoming its clinical limitations and simultaneously improving its efficacy [79]. The multifunctional nanoparticles significantly inhibited abnormal proliferation of fibroblast-like synoviocytes by scavenging reactive oxygen species and inhibiting secretion of proinflammatory cytokines. In adjuvant-induced arthritis rats, the half-life of circulation and levels of accumulated drugs at arthritic sites were obviously increased after treated by the nanoparticles, which effectively protected the bone destruction of these rats. Neuroinflammation induced by activated microglia/macrophages at injury sites in these patients can lead to brain blood barrier dysfunction, neuronal damage, and long-term neuronal and behavioral deficits. A hydroxy-capped fourth-generation polyamidoamine (PAMAM) dendrimer was used to encapsulate SIN for the treatment of traumatic brain injury, which selectively delivers SIN to activated microglia/macrophages at the site of injury and inhibits the deleterious effects of acute inflammation [80]. This conjugate can be rapidly taken up by cells, which increases the intracellular availability of SIN and significantly attenuates early/acute inflammation by inhibiting inflammatory factors, including TNF-α, IL-1β, CCL-3, and IL-6. They also decreased oxidative stress caused by iNOS and NO in lipopolysaccharides-activated mouse macrophages RAW 264.7 by inhibiting NF-κB activation and its nuclear translocation. *In vivo* imaging of a rabbit traumatic brain injury-controlled cortical impact model demonstrated that intravenous conjugates selectively targeted activated microglia/macrophages at the site of injury. A single intravenous injection of the conjugate effectively reduced inflammation caused by the expression of inflammatory cytokines at the injury site.

## 5. Other Delivery Systems

In addition to the extensively studied delivery systems described above, SIN-HCl has also been developed for ocular delivery and vaginal delivery to treat a variety of diseases. *In situ* gels using carbopol 940 and hydroxypropyl methylcellulose were prepared to contain SIN-HCl for the treatment of uveitis, which showed certain sustained release effect *in vitro* [81]. SIN was absorbed through the cornea *in vivo*. Pharmacokinetic study of SIN in the *in situ* gels and the control revealed their C_max_, half-life, AUC, and average retention time were 0.27 and 0.15 μg/mL, 81.64 and 65.94 min, 36.27 and 13.46 μg·mL^–1^·h, 124.16 and 100.89 h, respectively. This result suggests that *in situ* gel can improve the local bioavailability of SIN for ocular administration and achieve a continuous release of SIN. Vaginal administration is a method of parenteral administration, which can be used for the systemic administration of drugs with gastrointestinal degradation or severe liver first-pass effects [82]. SIN-loaded *in situ* gels with phytantriol as matrix was developed for vaginal administration [83], showing low viscosity, good fluidity, and non-irritating properties. They could be converted into high-viscosity cubic liquid crystal gel with a small amount of vaginal fluid and remain in the vagina for more than 12 h. SIN from this gel was released for 144 h, which was significantly prior than that of SIN-HCl solution (8 h) and carbomer gel (12 h).

## 6. Perspective

A variety of drug delivery systems of SIN have been developed to improve its poor pharmacokinetic properties and alleviate its side effects, considering its multifaceted mode of actions for RA and other disorders. A few delivery systems not only achieved sustained release in simulated *in vitro* models, but showed superior pharmacokinetic properties and therapeutically efficacy over control groups. Future clinical studies using some of these novel delivery systems may be expected. However, many SIN-based drug delivery systems have mainly focused on their physicochemical characterization and *in vitro* drug release. Future animal studies are needed to evaluate the pharmacokinetics and pharmacodynamics of each delivery system, as well as its therapeutic effects.

Due to the rise of solid chemistry and the facile approaches of solid preparations, more co-amorphous or co-crystal forms of SIN may be obtained by rational design and selection of drug co-formers to achieve sustained release. In addition, drug combination of SIN with methotrexate has shown increased efficacy for RA treatment; this suggests the great potential for drug delivery of SIN and methotrexate or other remedies together to achieve superior synergistic effects. The recent revelation of promising biological activities of SIN, such as anti-tumor, analgesic, cardiac protection, etc., means it is necessary to develop a series of alternative delivery systems for SIN for the treatment of other diseases beyond RA, and overcome many of the previous encountered side effects associated with RA treatment. In addition, many SIN derivatives are being prepared to improve its potency and achieve excellent therapeutic effects, some of them may also need targeted and effective delivery approaches. More in-depth and systematic research on multiple SIN drug delivery systems will help to achieve its full promise and minimize its side effects for the majority of RA patients. 

## Data Availability

Not applicable.

## Abbreviations

AA: adjuvant-induced arthritis; AS-TE, antioxidant ascorbic acid; AUC0-t, the area under the drug concentration–time curve from time zero to t; CbR, rhodamine B-loaded carbomer gel; CbS, sodium fluorescein-loaded carbomer gel; ChP, Chinese Pharmacopeia; Cmax, maximum concentration; CSF/Cplasma, SIN concentration in synovial fluid vs. that in plasma; Css, *in vivo* steady-state blood concentration; DMARDs, diseases-modifying antirheumatic drugs; DSS, dextran sulfate sodium; DTFS, deoxycholate edge activated transfersomes; ET, ethanol; H&E, Hematoxylin-Eosin; ISH2, *in situ* hexagonal liquid crystal; MMPTs, monoterpene edge activated PEGylated transfersomes; NSAIDs, non-steroidal anti-inflammatory drugs; PAMAM, polyamidoamine; PBS, phosphate buffered saline; PLO, pluronic lecithin organogel; PT, phytantriol; QR, rhodamine B-loaded cubic LC gel; QS, sodium fluorescein-loaded cubic LC gel; TE, transsethosome; TFSs, Transfersomes; RA, rheumatoid arthritis; SE, SIN-loaded ethosomes; SH-DM, SIN-loaded dissolving microneedle; SH-G, SIN gel; SIN, sinomenine; SIN-HCl, sinomenine-hydrochloride; VEA, vitamin E acetate.
